# Ndufaf2, a protein in mitochondrial complex I, interacts *in vivo* with methionine sulfoxide reductases

**DOI:** 10.1080/13510002.2023.2168635

**Published:** 2023-02-04

**Authors:** Sujin Park, José A. Trujillo-Hernandez, Rodney L. Levine

**Affiliations:** Laboratory of Biochemistry, National Heart, Lung, and Blood Institute, National Institutes of Health, Bethesda, MD, USA

**Keywords:** Methionine sulfoxide reductase, Ndufaf2, mimitin, TurboID proximity labeling, oxidative stress, methionine oxidation, mass spectrometry, mitochondrial complex I

## Abstract

**Background:**

Methionine sulfoxide reductases are found in all aerobic organisms. They function in antioxidant defense, cellular regulation by reversible oxidation of methionine in proteins, and in protein structure. However, very few *in vivo* binding partners or substrates of the reductases have been identified.

**Methods:**

We implemented a proximity labeling method, TurboID, to covalently link mitochondrial methionine sulfoxide reductase A (MSRA) to its binding partners in HEK293 cells. Proteomic analyses were performed to identify putative binding partners.

**Results:**

We show that human Ndufaf2, also called mimitin, is a binding partner of MSRA as well as all 3 MSRBs. We found that both methionine residues in Ndufaf2 were susceptible to oxidation by hydrogen peroxide and that the methionine sulfoxide reductases can reduce these methionine sulfoxide residues back to methionine.

**Conclusion:**

Methionine sulfoxide reductases can reduce methionine sulfoxide back to methionine in Ndufaf2. In addition to a repair function, it also creates a mechanism that could regulate cellular processes by modulation of methionine oxidation in Ndufaf2.

## Introduction

Within cells, mitochondria generate approximately 90% of reactive oxygen species (ROS) [1]. Antioxidant systems can efficiently scavenge ROS, but if the rate of generation of ROS exceeds the capacity of the defense system, oxidative damage may affect multiple cellular components, including DNA, protein, and lipids and can cause cell death [2]. Within proteins, the sulfur containing amino acids, cysteine and methionine, are notably sensitive to oxidation, both reversible and irreversible [3,4]. Enzyme systems have evolved both to protect these residues from irreversible oxidation and to utilize reversible oxidation as an oxidative defense as well as in cell signaling and regulatory mechanisms. Methionine sulfoxide reductases are present in all aerobic organisms and reduce methionine sulfoxide back to methionine. Oxidation of methionine to the sulfoxide introduces a chiral center, and reductases are specific for either S-methionine sulfoxide or R-methionine sulfoxide [5]. Mammals have one reductase specific for the S-epimer, MSRA, and three that are specific for the R-epimer, MSRB1, MSRB2, and MSRB3 [6]. Despite the ubiquitous presence of these reductases, only a few *in* vivo binding partners or substrates have been identified. These include actin [7,8], parkin [9], ataxin-2 [10], Fes1 [11], pyruvate kinase [12], STAT2 [13], STARD3 [14], and VAPA [15]. Because no *in vivo* binding partners for the mitochondrial form of MSRA had been identified, we applied proximity labeling to capture interacting proteins [16]. Proximity labeling of mitochondrial MSRA captured Ndufaf2, a protein important in the proper assembly of mitochondrial Complex I. We confirmed that Ndufaf2 interacts with MSRA and also show that it interacts with all 3 MSRBs. The reductases do reduce methionine sulfoxide residues of Ndufaf2 back reduced back to methionine. The interaction of the reductases with Ndufaf2 provides a repair function for Ndufaf2, and it may also provide a regulatory mechanism by which Ndufaf2 affects cellular metabolism.

## Materials and methods

### Antibodies and reagents

The monoclonal mouse anti-FLAG antibody (TA50011) and the polyclonal rabbit turboGFP (TA150071) for immunoprecipitation were obtained from Origene. The monoclonal mouse anti-FLAG (14793) for immunoprecipitation and immunofluorescence was purchased from Cell Signaling Technology. Polyclonal rabbit anti-Ndufaf2 antibody (13891-1-AP) was obtained from Proteintech. The rabbit polyclonal antiserum raised against our recombinant mouse MSRA was produced by Biomolecular Technology, Frederick, MD [17]. Streptavidin was detected by IRdye800 streptavidin (926-32230) from LI-COR. The secondary antibody for IRDye 680RD or 800CW goat anti-mouse (926-68070, 926-32210) and goat anti-rabbit (926-68071,926-32211) were purchased from LI-COR. Hydrogen peroxide (95321), tert-Butyl hydroperoxide (458139), and biotin (B4501) were obtained from Millipore Sigma.

### Cell culture and transfection

HEK293 and HeLa cells were purchased from ATCC. Cells were cultured under 5% CO_2_ at 37° in Dulbecco’s modified Eagle’s medium (DMEM) with high glucose (Thermo Fisher Scientific, 11995040) supplemented with 10% fetal bovine serum (FBS) (Thermo Fisher Scientific, A3160502) and 1% penicillin and streptomycin (Thermo Fisher Scientific, 10378016). For immunoprecipitation, HEK293 cells were transfected using calcium phosphate (Invitrogen, K278001) and for immunofluorescence, HeLa cells were transfected using Lipofectamine 3000 (Invitrogen, L3000008). Cells were provided fresh media for 2 h before transfection.

### DNA constructs

A human MSRA-TurboID plasmid was generated using the full length MSRA-GFP plasmid from Origene (RG208916). Using NotI and FseI restriction sites of the full length MSRA -GFP plasmid, GFP was exchanged with FLAG-tagged To provide a negative control, MSRA-TurboID was replaced by FLAG-tagged TurboID using the HindIII and FseI sites. FLAG-Ndufaf2 (Origene, RC207387) and human MSRBs tagged with turboGFP were purchased from Origene. Mutation of the 2 methionine residues of Ndufaf2 to valine was accomplished with the QuickChange XL Site-Directed mutagenesis kit (Agilent, 200516).

### Immunoprecipitation assays

HEK293 cells were co-transfected with FLAG-Ndufaf2 and human MSRA-turboGFP on 10 cm plates for 24 h and then lysed with IP lysis buffer (Thermo Fisher Scientific, 87787) which is composed of 25 mM Tris, pH 7.4, 150 mM NaCl, 1 mM EDTA, 1% NP-40, 5% glycerol, 1 mM PMSF, and 1X protease inhibitor. Lysates were cleared by centrifugation for 20 min at 21,100 g at 4° and then incubated with 3 μg of mouse anti-FLAG antibody (Origene, TA50011) for 2 h at room temperature. Lysate-antibody complexes were precipitated with 50 μl of Dynabeads proteinA (Invitrogen, 10002D) for 30 min at room temperature. The beads were then washed 3 times with IP lysis buffer and proteins were eluted by adding 100 ul of 5X SDS loading buffer and heating at 99° for 5 min. Immunoprecipitated proteins were separated by electrophoresis on 10∼20% Tris glycine gels (Invitrogen, XP10205BOX) and transferred to nitrocellulose membrane (Bio-Rod, 1704158). Membranes were probed with the indicated antibodies and quantitatively measured by an Odyssey infrared scanner Clx (LI-COR Biosciences).

### Production of his-tagged human Ndufaf2 recombinant protein

A his-tagged human Ndufaf2 plasmid was constructed in a pETDuet-1 vector with EcoRI and NotI restriction enzymes. Recombinant his-tagged Ndufaf2 wild type and methionine to valine proteins were prepared as described [18]. IPTG induced cell lysates were cleared by centrifugation for 30 min at 21,100 g. Cell lysates were incubated on a rotating platform at 4° for overnight with 5 ml of Ni-NTA Agarose (QIAGEN, 30210) after washing the beads 3 times with His-washing buffer containing 50 mM Tris, pH 8.0, 300 mM NaCl, 20 mM Imidazole, 0.1 mM EDTA, and 1 mM PMSF. Beads were loaded into a column and the flow-through collected. Then the beads were washed with 150 ml of wash buffer (Tris, pH 8.0, 50 mM NaCl, 20 mM Imidazole, 0.1 mM EDTA, and 1 mM PMSF), after which the column outflow was closed off. Thirty ml elution buffer (Tris, pH 8.0, 50 mM NaCl, 300 mM Imidazole, 0.1 mM EDTA, and 1 mM PMSF) was added, and the column held at room temperature for 10 min. The outflow was opened and 1 ml fractions were collected. Gel electrophoresis of 10 µl from each fraction was performed, followed by staining with Coomassie Blue. Fractions with the correct size of Ndufaf2 were dialyzed overnight against Tris, pH 8.0, 1 mM DTPA at 4°. The exact mass of the purified Ndufaf2 was determined by HPLC-MS as described [14].

### Immunofluorescence assay

Cells (5 × 10^4^/well) were plated onto poly-L lysine coated coverslips in a12-well plate. For visualization of the mitochondria or cytosol, cells were transfected with pCDNA-RFP Mito or pCDNA-RFP and fixed for 10 min at room temperature with 4% paraformaldehyde in phosphate buffered saline (PBS pH7.4, with a composition of 137 mM NaCl, 2.7 mM KCl, 8 mM Na_2_HPO_4_, and 2 mM KH_2_PO_4**)**_. Fixed samples were blocked with PBS containing 5% horse serum, 0.1% Triton X-100, and 0.05% NaN_3_ and then incubated overnight in a cold room with antibody to FLAG (1:500 dilution in blocking solution). Cells were washed three times with PBS and then incubated for 1 h at room temperature with an Alexa Fluor 488-conjugated secondary antibody (1:500 in blocking solution, Invitrogen) and Alexa Fluor 647-conjugated streptavidin (1:1000 in blocking solution, Invitrogen). Cells were mounted on a slide with ProLong Gold Antifade mounting solution (Invitrogen, P36962) containing 4′,6-diamidino-2-phenylindole (DAPI). Fluorescence images were captured with a Zeiss LSM 780, and image analyses were performed with ZEN software, black edition (Zeiss).

### Sample preparation for proteomics analysis

MSRA-TurboID and Non-targeting TurboID were transfected into HEK293 cells in 15 cm cell culture dishes using calcium phosphate. They were incubated for 48 h after which 50 μM biotin was added and incubation continued another 3 h. Cells were lysed with RIPA buffer and an aliquot containing 4 mg protein (BCA assay) was taken. 300 μl streptavidin beads were washed three times with RIPA buffer. The lysate aliquot was added, and the beads were incubated at 4° overnight. Then additional bead washing and trypsin digestion were performed as described [16]. After overnight digestion, samples were acidified by adding 1 μl of 10% trifluoroacetic acid. The digests were desalted with C18-loaded pipette tips (Pierce, 87784). To measure the peptide content with a colorimetric peptide assay (Pierce, 23275), 120 μg of peptides were taken from each sample and dried by vacuum centrifugation.

### Liquid chromatography-tandem MS analysis

Desalted tryptic peptides were resuspended in 2% acetonitrile/0.01% formic acid and analyzed by nanoscale liquid chromatography-tandem mass spectrometry (nLC- MS/MS). The instrument was an Ultimate 3000-nLC online coupled with an Orbitrap Lumos Tribrid mass spectrometer (Thermo Fisher Scientific). Peptides were separated on an EASY-Spray C18 column (Thermo Fisher Scientific, 75 μm by 50 cm inner diameter, 2-μm particle size, and 100-Å pore size). Separation was achieved by a 4–32% linear gradient of acetonitrile with 0.1% formic acid over 90 min. An electrospray voltage of 1.9 kV was applied to the eluent via the EASY-Spray column electrode. The Orbitrap Lumos was operated in positive ion data-dependent mode. Full-scan MS was performed in the Orbitrap with a normal precursor mass range of 375–1500 *m/z* (mass/charge ratio) at a resolution of 120,000. The automatic gain control (AGC) target and maximum accumulation time settings were set to 4 × 10^5^ and 50 ms, respectively. MS2 was triggered by selecting the most intense precursor ions above an intensity threshold of 2.5 × 10^4^ for higher energy collisional dissociation (HCD)–MS2 fragmentation with an AGC target and maximum accumulation time settings of 5 × 10^4^ and 54 ms, respectively. Mass filtering was performed by the quadrupole with 1.2 *m/z* transmission window, followed by HCD fragmentation in the orbitrap at a resolution of 15,000 and collision energy of 32%. To improve the spectral acquisition rate, parallelizable time was activated. The number of MS2 spectra acquired between full scans was restricted to a duty cycle of 3 s. Raw data files were processed with the Proteome Discoverer software (v2.4, Thermo Fisher Scientific), using Sequest HT (Thermo Fisher Scientific). The following search parameters were set: protein database UniProtKB/Swiss-Prot *Homo Sapiens* (20,308 sequences release 2021_01) concatenated with reversed copies of all sequences; MS1 tolerance of 12 ppm: orbitrap detected MS/MS mass tolerance of 0.02 Da; enzyme specificity set as trypsin with maximum two missed cleavages; minimum peptide length of 6 amino acids; fixed modification of Cys (carbamidomethylation); variable modification of methionine oxidation and acetyl on N terminus of protein. Percolator algorithm (v.3.02.1, University of Washington) was used to calculate the false discovery rate (FDR) of peptide spectrum matches (PSM), set to a q-value <0.05 [19–22].

### Statistical analysis

Quantitative data are presented as mean ± standard deviation, and significance was tested with Student’s t-test. *P* < 0.05 was considered statistically significant.

## Results

### Optimization of MSRA-TurboID HEK293 cells

**Figure 1.**
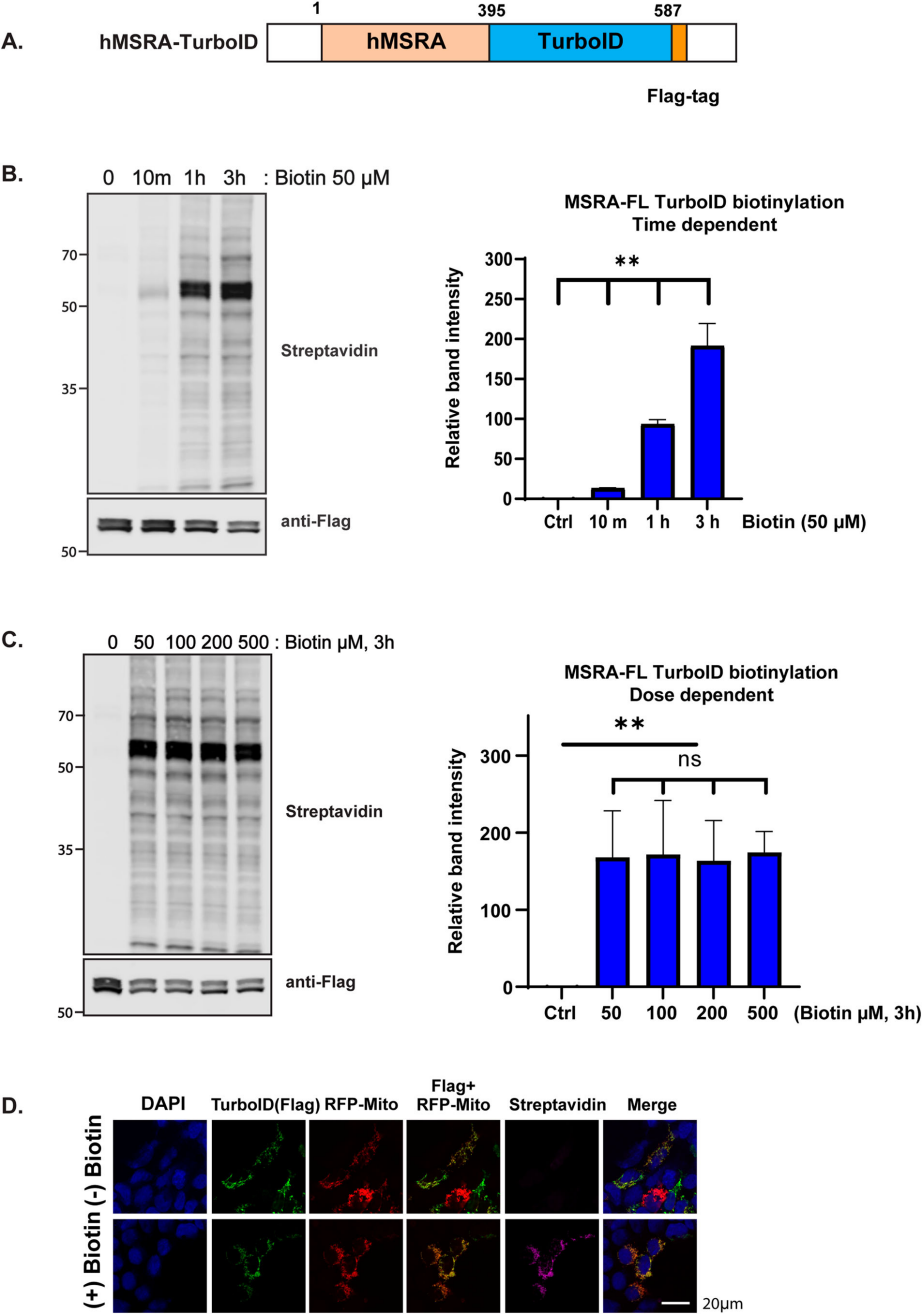
Optimization of TurboID biotinylation time and concentration and demonstration of its mitochondrial localization. (A) Diagram of the expression cassette for the expression of the biotin ligase. Human MSRA was fused to the N-terminus and a FLAG tag was added to the C-terminus of TurboID. (B) Time course of TurboID–based proximity labeling with 500 µM biotin in HEK293 cells. Streptavidin-800 and anti-FLAG antibodies were used for detection of biotinylated proteins (top panel) and the loading control (bottom panel). The ratio of the intensity of streptavidin-800 to anti-FLAG is plotted in the graph on the right. The results shown are means ± SD with *n* = 3. (C) Biotin concentration dependence of labeling. HEK293 cells were labeled for 3 h and analyzed as in panel B, again with *n* = 3. (D) Confocal microscopy of fluorescence imaging of HeLa cells co-overexpressing MSRA-TurboID and RFP-Mito as a mitochondrial marker. Cells were either untreated or incubated with 100 µM biotin for 10 min. They were stained with anti-FLAG antibody to visualize MSRA-TurboID and with streptavidin-647 to visualize biotinylated proteins. In all panels, statistically significant differences between treatments were marked with asterisks (*p < 0.05; **p < 0.01).

We fused the human MSRA to the N-terminus of TurboID and placed a FLAG tag at the C-terminus (Figure 1(A)). The time course of biotinylation with 500 µM biotin is shown in (Figure 1(B)). Labeling occurred by 10 min and increased at 1 and 3 h. (Figure 1(C)) shows the dependence of labeling on the biotin concentration. We found that 50 µM biotin was sufficient to give maximal biotinylation at 3 h, and we used this time and biotin concentration routinely. The MSRA gene can produce proteins that are localized to either the cytosol or the mitochondria through initiation at 2 different sites [23], but immunofluorescence imaging established that the MSRA-TurboID protein was localized to mitochondria (Figure 1(D)).

### Identification of Ndufaf2 as a binding partner of MSRA

**Figure 2.**
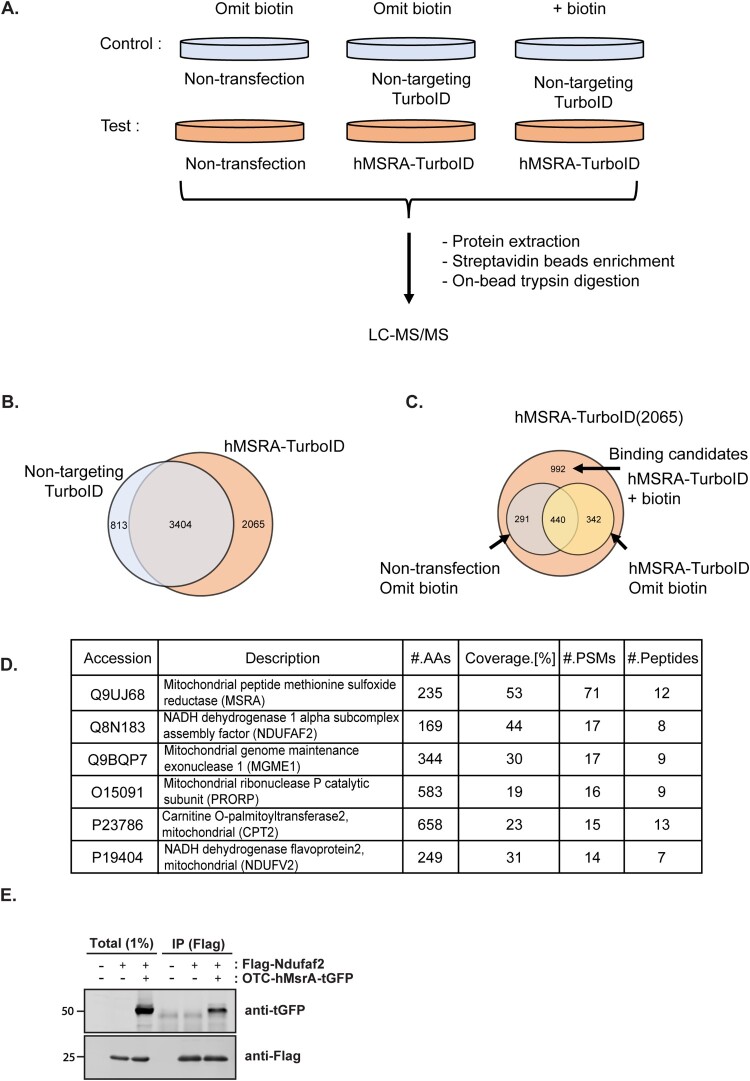
Proteomics data analysis and identification of Ndufaf2 as a binding partner of mitochondrial MSRA. (A) The experimental design for proteomic analysis of proximity labeling of mitochondrial MSRA. binding partner. HEK293 cells were incubated with 50 µM of exogenous biotin for 3 h. The HEK293 cells expressed either a non-targeting TurboID or the targeted MSRA-TurboID. (B) First filtration step: Removal of proteins present in both targeted and non-targeted cells incubated with biotin leaves 2,065 proteins found only in the targeted cells. (C) Second filtration step: From the 2,065 proteins remaining after the first filtration, we removed those that were present in the non-transfected controls and those present without biotin incubation. This filtration reduces the candidate list to 992 proteins. (D) Proteins top ranked by peptide spectrum matches (PSMs) in the proteomic analysis. (E) Co-immunoprecipitation of mitochondrially-targeted MSRA and Ndufaf2. HEK293 cells co-expressed FLAG-tagged Ndufaf2 and mitochondrially targeted MSRA tagged with tGFP at its C-terminus. Cell lysates were immunoprecipitated with anti-FLAG antibody and detected by anti-tGFP antibody.

A scheme of the experimental design is shown in (Figure 2(A)). We constructed a mitochondrial-targeted MSRA-TurboID vector and, as a control, a non-targeted MSRA-TurboID that lacks the mitochondrial targeting sequence. For both constructs, we analyzed cells of a non-transfected control incubated without biotin, a transfected control incubated without biotin, and a transfected sample incubated with biotin.

The proteomic results for the targeted, biotin-treated cells were filtered to remove proteins that were also present in the non-targeted control, leaving 2,065 proteins (Figure 2(B)). A second filtration was applied to remove proteins present in the non-transfected control and the control not incubated with biotin. This left 992 proteins that were biotin-dependent and present only in the targeted cells (Figure 2(C)). We then ranked the 992 binding candidates by the number of nonpeptide spectral matches (PSMs) in the proteomic analysis (Figure 2(D)). Of course, the highest number of matches was to MSRA, followed by NADH Ubiquinone Oxidoreductase Complex Assembly Factor 2 (Ndufaf2), a protein important to the correct assembly of the mitochondrial Complex I. To assess whether this candidate protein actually interacted *in vivo* with mitochondrial MSRA, we created a mitochondrial targeted MSRA plasmid by fusing the very strong mitochondrial targeting sequence of ornithine carbamoyltrasferase to the N-terminus of MSRA. This mitochondrially targeted MSRA was co-expressed with FLAG-tagged Ndufaf2 in HEK293 cells. MSRA was co-immunoprecipitated with Ndufaf2. (Figure 2(E)), confirming that Ndufaf2 interacts with mitochondrial MSRA.

### Methionine residues of Ndufaf2 protein are oxidized by reactive oxygen species

**Figure 3.**
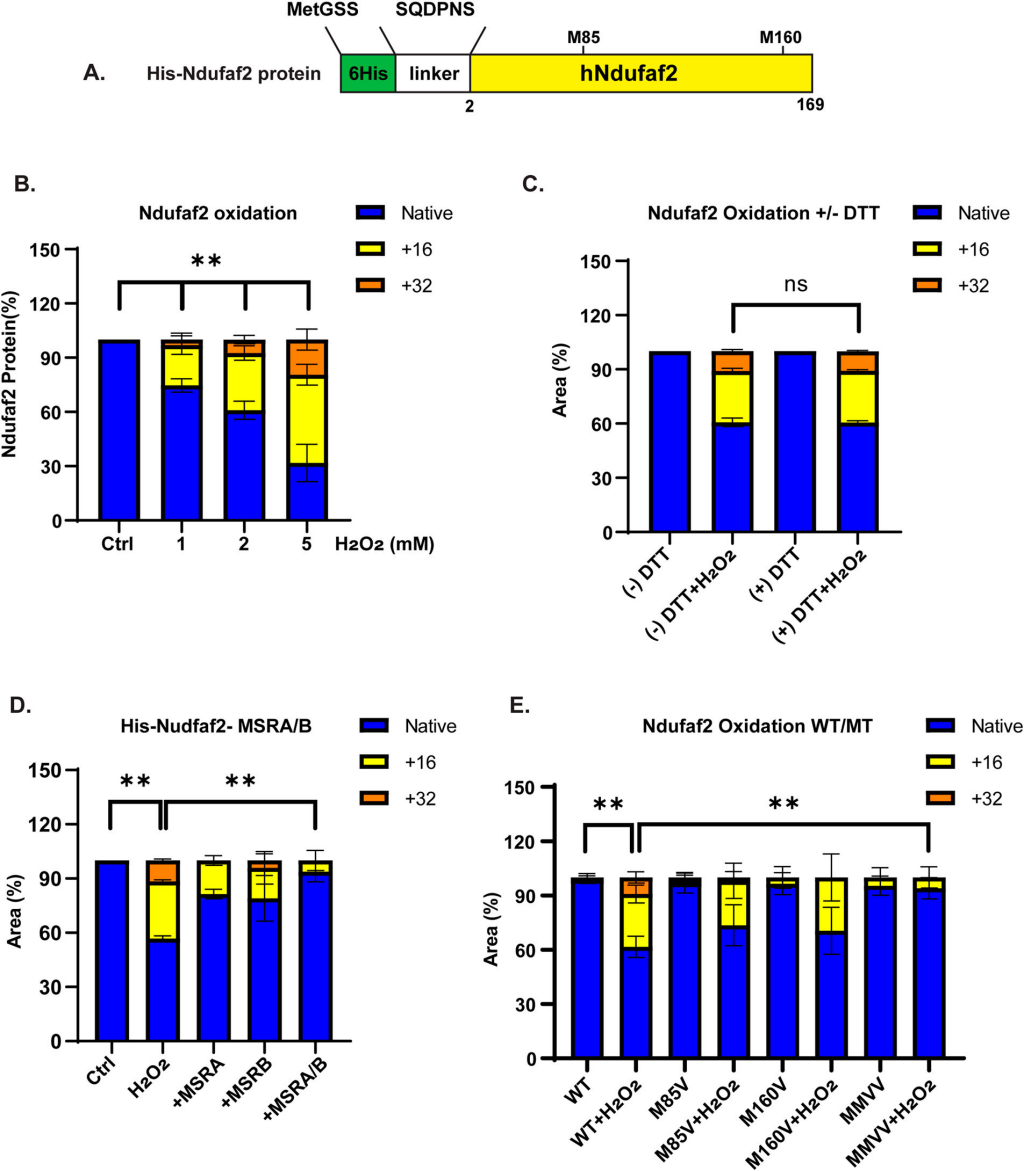
Hydrogen peroxide oxidizes both methionines in Ndufaf2 and MSRs reverse the oxidation. (A) Diagram of His-tagged human Ndufaf2. (B) Ndufaf2 oxidation by hydrogen peroxide. Ndufaf2 was incubated with the indicated concentration of hydrogen peroxide for 1 h at 37°. The fraction oxidized was measured by mass spectrometry. An increase of 16 Da (gray bar) is due to conversion of 1 methionine to methionine sulfoxide. An increase of 32 Da (black bar) is due to conversion of 2 methionines to methionine sulfoxide or of 1 methionine to methionine sulfone. MSRs cannot reduce the sulfone, so the results in panel C establish that the +32 Da is due to conversion of 2 methionines to their sulfoxide. Results are given as the mean and SD for 3 separate experiments. (C) DTT alone cannot reduce oxidized Ndufaf2. Samples prepared as in panel B and then incubated with 10 mM DTT for 10 min. (D) MSR reduce oxidized Ndufaf2. Ndufaf2 was incubated at 37° for 1 h with or without 2 mM of hydrogen peroxide. Residual hydrogen peroxide was removed by incubating with DTT for 10 min. Then 4 μM of MSRA and of MSRB were added and incubation continued for 30 min. The reaction was stopped by making the solutions 0.5% in trifluoroacetic acid. Results are given as the mean and SD for 3 separate experiments. (E) Native and mutant Ndufaf2 susceptibility to oxidation by hydrogen peroxide. Samples were prepared and analyzed as in panel B. MMVV is the M85 V/M160 V mutant. Results are given as the mean and SD for 3 separate experiments.

**Figure 4.**
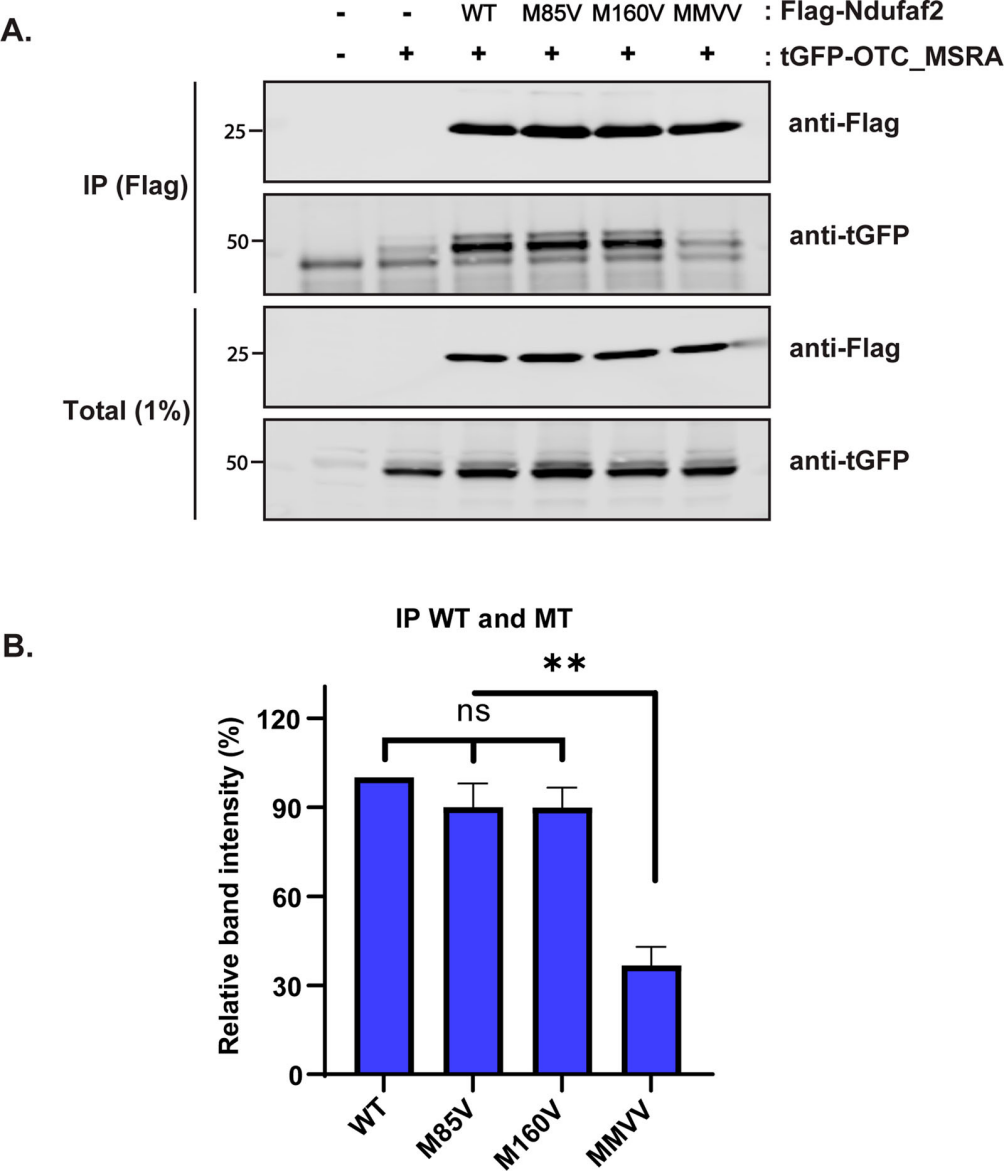
Interaction of Ndufaf2 with methionine mutants. (A) Coimmunoprecipitation of Ndufaf2 and MSRA. Mitochondrially targeted tGFP-tagged MSRA was expressed in HEK293 along with FLAG-tagged Ndufaf2. Lysed cells were immunoprecipitated with anti-FLAG antibody and probed for Nduafa2 with anti-tGFP antibody. (B) Quantitation of Ndufaf2 coimmunoprecipitated with MSRA. The intensities of the bands in panel (A) were quantitated with the Odyssey infrared scanner. Results are given as the mean and SD for 3 separate experiments.

We then investigated whether Ndufaf2 is oxidized by reactive species such as hydrogen peroxide using His-tagged human Ndufaf2 protein (Figure 3(A)). The fraction of protein oxidized increased during a 1 h incubation with hydrogen peroxide, reaching 70% when the peroxide concentration was 5 mM (Figure 3(B)). We added 10 mM DTT and incubated 10 min to remove the remaining hydrogen peroxide. DTT alone cannot reverse the oxidation (Figure 3(C)). Incubation with DTT along with MSRA or MSRB reduced the oxidized fraction to 20%, and incubation with both MSRA and MSRB almost completely reversed oxidation (Figure 3(D)). The MSR are specific for methionine sulfoxide, and therefore the reversal of oxidation established that hydrogen peroxide only oxidized methionine in Ndufaf2. Human Ndufaf2 has 2 methionine residues, Met85 and Met160. To assess their individual susceptibility to oxidation, we produced mutants in which one or both were changed to valine. Both M85 V and M160 V were equally susceptible to oxidation while the double mutant was resistant, as expected (Figure 3(E)). Ghesquière and colleagues reported that hydrogen peroxide exposure of Jurkat cells caused oxidation of Met85, although they did not detect oxidation of Met160 in their proteomics study [24]. Co-immunoprecipitation of Ndufaf2 and MSRA was equivalent to wild-type with the two single mutants, but decreased substantially with the double mutant (Figure 4(A,B)).

### All 4 human MSR interact with Ndufaf2

**Figure 5.**
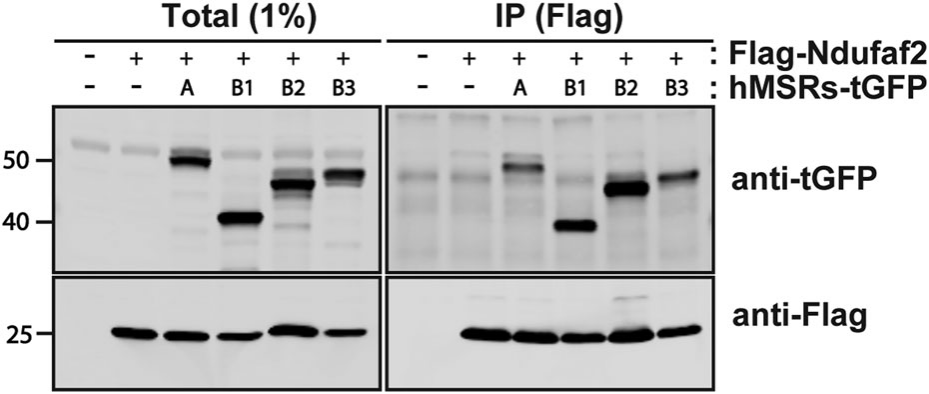
All 4 human MSR interact with Ndufaf2. FLAG-tagged Ndufaf2 and tGFP-tagged MSRA or MSRBs were overexpressed in HEK293 cells. Lysed cells were immunoprecipitated with anti-FLAG antibody and probed for Nduafa2 with anti-tGFP antibody.

The complete reduction of oxidized Ndufaf2 demonstrated in (Figure 3(C)) was accomplished with recombinant human MSRA and *E. coli* MSRB. We thus investigated which of the 3 human MSR interacted with Ndufaf2 *in vivo* and found that all 3 co-immunoprecipitate with Ndufaf2 (Figure 5). Thus, all 4 human reductases interact with Ndufaf2, just as all 4 interact with the cholesterol transporting protein STARD3 and the endoplasmic reticulum-lysosome membrane contact site protein VAPA [15].

## Discussion

We utilized proximity-labeling to identify the mitochondrial complex I protein, Ndufaf2, as a binding partner of all 4 methionine sulfoxide reductases. We also showed that the 2 methionines of Ndufaf2 can be oxidized to methionine sulfoxide and reduced back to methionine by the combined action of MSRA and MSRB. Ndufaf2 was first identified as a myc-controlled gene involved in proliferation of an esophageal carcinoma [25] and was named mimitin (Myc-induced mitochondrial protein). Both the transcript and protein are upregulated by the proinflammatory cytokine IL-1 [26]. Studies in yeast and cells from a child with a progressive neurodegeneration provided evidence than Ndufaf2 is a chaperone for assembly of Complex I [27]. However, a subsequent study showed that Ndufaf2 is dispensable for assembly of Complex I, although assembly was slower in Ndufaf2 deficient cells [28]. Notably, that study also demonstrated that Ndufaf2 deficiency caused oxidative stress and mitochondrial DNA deletion. The authors proposed that Ndufaf2 is a chaperone that assists in the proper folding of proteins within Complex I, rather than functioning as a classical assembly factor (Figure 5).

Mitochondria produce most of the reactive oxygen species of the cell [1,29]. Thus, Ndufaf2 functions in an oxidizing environment, and we show that the two methionines in Ndufaf2 are subject to oxidation to methionine sulfoxide. However, a limitation of this study is that we performed experiments with 1 mM hydrogen peroxide. While that is a supra-physiological concentration [30], it is experimentally practical because of the kinetics of reaction of hydrogen peroxide with methionine. The rate constant for reaction of methionine with peroxide is orders of magnitude less than the rate constant for reaction with thiols [31,32]. Thus, relatively high concentration of peroxide must be used in order to have a rate that is practical. All 4 human MSR bind to Ndufaf2 and are able to effectively reduce the sulfoxides back to methionine. Any Ndufaf2 molecules that have been damaged by reactive species can be repaired and continue to function as a chaperone in Complex I assembly. Failure of repair might lead to misfolded proteins, increased oxidative stress, and mitochondrial DNA deletion as occurs in experimentally induced Ndufaf2 deficiency [28]. In addition, regulated oxidation and reduction of methionine residues is now recognized as a mechanism of cellular regulation [4], and methionine-oxidized Ndufaf2 could have this function as well.

## Data Availability

Detailed mass spectrometry data, including the raw data files, and raw imaging files are available upon request. All other data are in the manuscript.
